# Charting a path to end the AIDS epidemic

**DOI:** 10.2471/BLT.16.176875

**Published:** 2016-06-01

**Authors:** Michel Sidibé

**Affiliations:** a Joint United Nations Programme on HIV/AIDS, avenue Appia 20, 1211 Geneva 27, Switzerland.

From 8 to 10 June 2016, heads and representatives of states and governments, along with other key stakeholders, will assemble at the United Nations (UN) in New York, for the High-Level Meeting on Ending AIDS. There are three reasons why this meeting is an important milestone for the global response against human immunodeficiency virus (HIV) and acquired immunodeficiency syndrome (AIDS). 

First, the meeting will provide an opportunity to reflect on the extent of progress and unprecedented achievements that have been made in responding to the AIDS epidemic, as described in the UN Secretary-General’s report.^1^ Globally, between 2000 and 2014, the number of people newly infected with HIV each year dropped by more than one-third, from 3 million to 2 million. In the same period, new paediatric HIV infections fell by 58%. Eighty-five countries are close to eliminating mother-to-child transmission of HIV. The global target of millennium development goal 6 of having 15 million people on HIV treatment by 2015 was met nine months ahead of schedule.^2^

Second, the meeting serves as an occasion to galvanize support from the global community to scale up the AIDS response. The 90–90–90 treatment target calls for 90% of people living with HIV to know their status, 90% of people who know their HIV status to have access to treatment and 90% of people on treatment to achieve suppressed viral loads by 2020.^3^ We need to focus on specific populations and locations where investments will have the greatest impact to ensure that no one is being left behind. Of the estimated 2 million people newly infected with HIV in 2014, nearly half live in eastern and southern Africa, where adolescent girls and young women are disproportionately at risk.^3^ The number of people acquiring HIV infections is increasing in several countries. Incident infections are also increasing in some cities in North America and western Europe. These increases in new infections are primarily seen in men who have sex with men, transgender people, sex workers and their clients and people who inject drugs. Across Latin America and the Caribbean, most cases occur in men who have sex with men.^1^ We have less than five years to step up investments in HIV prevention, treatment and care to reach this target. 

Third, the meeting will be an opportunity to reflect on specific challenges that need to be addressed as we move forward. Among them is a treatment gap and inadequate global investments in prevention.^4^ Other challenges include how to increase access to diagnostic tests and eliminate HIV-related discrimination. Around half of all people living with HIV are unaware of their HIV status.^1^ While there is a general decline in discriminatory attitudes towards people living with HIV, in about 40% of countries where studies have been conducted, more than half of adults surveyed reported discriminatory attitudes towards people living with HIV.^5^ Laws, policies and practices that are fuelling vulnerability to HIV and hindering access to services will need to be addressed as a priority.

The *UNAIDS 2016–2021* *Strategy *integrated efforts towards ending the AIDS epidemic fully into *Transforming our world: the 2030 agenda for sustainable development.* The strategy sets out the links between the HIV response and several sustainable development goals (SDGs), from SDG 1 on ending poverty to SDG 16 on promoting inclusive societies.^3^

Building on the SDGs, we need a global approach to ending the AIDS epidemic, underpinned by universal principles of social equity and an unwavering commitment to human rights. We must pledge to invest at least 26 billion United States dollars a year in low- and middle-income countries by 2020. Diverse sources of funding, innovative forms of financing and increases in both domestic public expenditure and international assistance are all needed.

Let us make history at the high-level meeting. Let us commit to charting a path forward that will secure the end of the AIDS epidemic as a public health threat in one generation.
